# Complete Genome Sequence of *Porcine respirovirus 1* Strain USA/MN25890NS/2016, Isolated in the United States

**DOI:** 10.1128/genomeA.01139-17

**Published:** 2017-10-19

**Authors:** Jie Yeun Park, Michael Welch, Karen M. Harmon, Jianqiang Zhang, Pablo E. Piñeyro, Ganwu Li, Phillip C. Gauger

**Affiliations:** Department of Veterinary Diagnostic and Production Animal Medicine, College of Veterinary Medicine, Iowa State University, Ames, Iowa, USA

## Abstract

A porcine parainfluenza virus type 1 (species *Porcine respirovirus 1*) cell culture isolate, USA/MN25890NS/2016, was obtained from porcine nasal swabs, and its complete genome sequence (GenBank accession number MF681710) was determined to help further characterize this virus.

## GENOME ANNOUNCEMENT

Porcine parainfluenza virus type 1 (PPIV-1) (species *Porcine respirovirus 1*) was first identified in 2013 from nasopharyngeal samples collected from deceased slaughterhouse pigs in Hong Kong, China. The PPIV-1 genome was reported at that time to be a novel paramyxovirus with highest genetic similarity to Sendai virus and human parainfluenza virus type 1 (1).

Porcine respirovirus type 1 is a member of the genus *Respirovirus* in the *Paramyxoviridae* family and is an enveloped, single-stranded, negative sense RNA virus (1). PPIV-1 is widespread in the United States and has been detected by real-time reverse transcription PCR (rRT-PCR) in all ages of swine but more commonly in nursery and grow-finish pigs. PPIV-1 has been detected in respiratory samples, including lung tissues, nasal swabs, and oral fluids that have been submitted to the Iowa State University Veterinary Diagnostic Laboratory (2). Viral infection has been associated with respiratory disease in pigs with clinical signs that may include coughing, minor sneezing, and serous nasal discharge (3). It has been suggested that PPIV-1 may play a role in the porcine respiratory disease complex (2). However, the pathogenesis of PPIV-1 has not been fully elucidated, and the causal links between PPIV-1 and disease have not been established. Obtaining a PPIV-1 isolate propagated in cell culture is critical for experimental challenge to evaluate the pathogenesis and clinical significance of viral infection in swine. In addition, the availability of a PPIV-1 isolate can help develop and validate virological and serological diagnostic assays and assist vaccine development.

Virus isolation was attempted using rRT-PCR-positive respiratory samples from random submissions with a history of respiratory disease. The virus was eventually isolated from nasal swabs and confirmed by observing a cytopathic effect in tissue culture, rRT-PCR analysis, and Sanger sequencing of the hemagglutinin-neuraminidase (HN) and fusion (F) genes.

The complete genome of PPIV-1 isolate USA/MN25890NS/2016 was acquired using next-generation sequencing (NGS) technology on an Illumina MiSeq platform and analyzed using the DNASTAR Lasergene 12 following previously described procedures (4, 5). The sequence consists of 15,334 nucleotides and encodes six open reading frames (3′-N-P-M-F-HN-L-5′). The HN and F genes are major glycoproteins on the viral surface suspected to play an important role in receptor binding, fusion, and penetration (6).

The full genome sequence of this PPIV-1 isolate is more closely related to PPIV-1 strains detected in the United States in 2016 (GenBank accession numbers KT749882 and KT749883), forming a clade with 98.1% and 98.2% nucleotide homology in a phylogenetic analysis using Clustal W in DNASTAR Lasergene 12. In contrast, the PPIV-1 whole genome demonstrated 91.3%, 95.9%, and 96% identities with virus strains detected in Hong Kong in 2013 (accession numbers JX857410, JX857409, and JX857411, respectively). These data suggest that the PPIV-1 virus is genetically evolving over time and at different geographic locations.

The association of this PPIV-1 sequence with clinical respiratory disease in swine remains unknown. Further analysis and experimental challenge are required to determine the clinical significance of this PPIV-1 isolate.

### Accession number(s).

The complete genome sequence of PPIV-1 isolate USA/MN25890NS/2016 has been deposited in GenBank under the accession number MF681710.
